# Prevalence and associated factors of obesity and overweight in Chinese patients with bipolar disorder

**DOI:** 10.3389/fpsyt.2022.984829

**Published:** 2022-09-06

**Authors:** Wenying Yi, Haibo Wu, Ruikeng Li, Haijing Li, Zhen Song, Shenglin She, Yingjun Zheng

**Affiliations:** The Affiliated Brain Hospital of Guangzhou Medical University, Guangzhou, China

**Keywords:** obesity, overweight, prevalence, risk factors, bipolar disorder

## Abstract

**Object:**

Despite abundant literature demonstrating a high prevalence of obesity and overweight in people with bipolar disorder (BD), little is known about this topic in China. Therefore, we assessed the prevalence and associated factors of obesity and overweight among inpatients with BD in our hospital, one of the largest public psychiatric hospitals in China.

**Methods:**

In this retrospective, cross-sectional study, 1,169 inpatients ≥18 years with BD during 2019 were included. Obesity was defined as having a BMI ≥25 kg/m^2^, and overweight was defined as having a BMI from 23 kg/m^2^ to <25 kg/m^2^. Binary logistic regression analysis was performed to identify factors associated with obesity and overweight.

**Results:**

The prevalence of obesity and overweight was 21.0% and 32.2% in patients with BD, respectively. Compared to patients with overweight and normal weight, patients with obesity were older, had a longer duration of BD and a longer length of hospital stay, had a higher prevalence of diabetes and hypertension, and had a higher level of all metabolic indices, except for HDL cholesterol. Binary logistic regression analysis showed that duration of BD, uric acid, alanine aminotransferase (ALT), triglyceride, and LDL cholesterol were significantly associated with obesity, and male sex and uric acid level were significantly associated with overweight (*p* < 0.05).

**Conclusions:**

Obesity and overweight were fairly prevalent in Chinese BD patients, and several factors were related to obesity and overweight. The results of the present study call for the need to implement early screening, prevention and interventions for obesity and overweight in patients with BD in China.

## Introduction

Bipolar disorder (BD) is one of the most severe and function-impaired mental disorders with a high global burden. BD affected 4.53 million people in 2017 (1). The disability-adjusted life years (DALYs) of BD increased by 54.4%, from 6.02 million in 1990 to 9.29 million in 2017 (1). In the United States, the estimated total annual national economic burden of BD/bipolar I disorder (BD-I) subtype was more than $195 billion, with ~25% attributed to direct medical costs (2). People with BD/BD-I used health-care services more frequently and had higher direct medical costs than matched controls (2). A meta-analysis with 31 studies suggested that people with BD had a 7.42-fold increased risk of unnatural death and a 1.64-fold increased risk of natural death compared to the general population (3). A 1.73 times risk of deaths from circulatory illnesses, 2.92 times risk of deaths from respiratory illness, 2.25 times risk of deaths from respiratory illness, and 1.14 times risk of deaths from neoplasm caused these natural deaths in patients with BD (3). Data from a meta-analysis of 32 studies between 1984 and 2013 with 470,411 participants showed that the pooled prevalence of BD was generally lower in China than in Western countries (4). Recently, a study based on a multistage, stratified, cluster random sampling method with 20,884 participants also found that the prevalence of BD was lower in China than in other countries (5). However, Chinese patients with BD also had higher all-cause, natural-cause and unnatural-cause mortality rates than the general population (6). A study reported that Chinese men and women with BD had 6.78 years and 7.35 years of excess life-years lost, respectively (6). Respiratory diseases, cardiovascular diseases and cancers accounted for the majority of deaths among Chinese BD (6).

In recent years, the prevalence of obesity has increased globally, and obesity has become a major public health problem (7). In 2010, it was estimated that overweight and obesity cause 3.4 million deaths, 3.9% of years of life lost, and 3.8% of DALYs worldwide (7). Based on Chinese criteria, the estimated Chinese national prevalence rates of obesity and overweight were 16.4 and 34.3% between 2015 and 2019, respectively (8). Economic developments, sociocultural norms, substantial changes in dietary patterns, decreased physical activity levels, increased sedentary behaviors, genetic susceptibility, psychosocial factors, obesogens, and *in utero* and early-life exposures drive the growing burden of overweight and obesity in China (8). A Chinese study found that the total, direct, and indirect costs of the four obesity-related illnesses were up to $30,350.8 million, $28,642.5 million, and $1,708.3 million, respectively, with 12.7% attributable to general obesity and 28.7% attributable to central obesity (9). Even in 2003, the estimated total medical cost attributable to overweight and obesity reached ~$2.74 billion, accounting for 25.5% of the total medical costs for the four obesity-related chronic diseases or 3.7% of the national total medical costs (10). If not eliminated, obesity is associated with an increased risk of diseases such as type 2 diabetes mellitus, fatty liver disease, hypertension, myocardial infarction, stroke, dementia, osteoarthritis, obstructive sleep apnea and several cancers, leading to a decline in both quality of life and life expectancy (11). Moreover, previous evidence has shown that obesity is closely correlated with an increased risk of developing mental disorders, including mood disorders, anxiety disorders, personality disorders, attention deficit hyperactivity disorder (ADHD), binge eating disorders, trauma, BD, and schizophrenia (12). Maternal obesity has been found to be linked with neuropsychiatric disorders, including ADHD, autism spectrum disorders, anxiety, depression, schizophrenia, eating disorders, and impairments in cognition in offspring (13). Metabolic alterations, systemic inflammation, oxidative stress, neuroinflammation and impaired brain plasticity induced by a high-fat diet have been found to be tightly interconnected processes, implicating the role of obesity in the pathogenesis of neurological diseases (14).

People with BD have a high prevalence of obesity. A meta-analysis of 49 studies found that the pooled prevalence of general obesity (BMI ≥ 30 kg/m^2^) was 29.0% among 322,494 adults with BD, which was significantly higher than their healthy counterparts (15). This meta-analysis also suggested that the pooled prevalence of abdominal obesity in 2,378 BD patients was up to 51.1%, and female BD patients and duration of BD were significantly associated with abdominal obesity (15). It has been reported that illness-related factors (mood-related factors, i.e., mania or depression), treatment-related factors (weight implications and other side effects of medications), and lifestyle factors (physical inactivity, poor diet, smoking, substance abuse) were associated with obesity among people with BD (16). Although current evidence remains controversial in most aspects of clinical outcomes, existing evidence suggests that obesity in BD places patients at considerable risk for poor outcomes, such as altering the course of BD, worsening global functioning, poor treatment response and a chronic course of illness, and enhancing rapid cycling (17). Furthermore, in young people with BD, obesity has been shown to be associated with physical abuse, suicide attempts, self-injurious behaviors, psychotropic medication, and psychiatric hospitalizations (18).

Despite the substantial number of studies assessing obesity and overweight in people with BD (15), little is known about obesity and overweight among Chinese patients with BD. To our knowledge, only two studies have assessed obesity in Chinese BD patients. One early prospective study of 148 Chinese adult patients with BD reported that the prevalence of obesity (BMI ≥25 kg/m^2^) increased from 34.5% at baseline to 45.3% at the study endpoint (19). Another recently published study found that the prevalence of obesity and overweight were 17.74 and 34.68% in 124 patients with stable BD, respectively (20). Both of these studies suggested that obesity was prevalent in Chinese patients with BD, but they did not investigate the associated factors of obesity and overweight and only included a small sample.

Therefore, we conducted this study to investigate the prevalence and associated factors of obesity and overweight in a larger sample of inpatients with BD in China. This study will add to the body of knowledge on obesity and overweight among BD patients from a large public psychiatric hospital in China.

## Methods

### Subjects and study design

This retrospective observational study was conducted at the Affiliated Brain Hospital of Guangzhou Medical University, which is one of the largest public mental health centers with 1,920 beds for inpatient service in China. The ethics committee of the Affiliated Brain Hospital of Guangzhou Medical University approved this retrospective cross-sectional study. The diagnosis of mental disorders was established by two experienced psychiatrists according to the 10th revision of the International Classification of Diseases (ICD-10).

The inclusion criteria were as follows: (1) inpatients diagnosed with BD; (2) aged ≥18 years; (3) admitted to our hospital between January 1, 2019, and December 31, 2019; and (4) had a record of BMI at admission to the hospital. The exclusion criteria were as follows: (1) inpatients aged <18 years; (2) severe physical diseases, pregnancy, lactation, or missing BMI data. If the patients were readmitted during 2019, only the data of the first admission for the patients were collected and used for analysis.

### Data collection and analysis

Data were extracted from the electronic databases of this hospital. The collected data were anonymized. The following data were collected for all of the included inpatients: sex, age, duration of BD, length of hospital stay, BMI at admission, and diagnoses at discharge. Laboratory results of the first blood tests were collected, including uric acid, alanine aminotransferase (ALT), triglyceride, total cholesterol, LDL cholesterol, and HDL cholesterol. The first blood tests are usually conducted on the second day after admission to our hospital.

### Definitions of obesity and overweight

Based on the Asian-specific cutoff points (21), patients with a BMI <23 kg/m^2^ were defined as normal weight, patients with a BMI from 23 kg/m^2^ to <25 kg/m^2^ were defined as overweight, and patients with a BMI ≥ 25 kg/m^2^ were defined as obese for both men and women.

### Data analysis

Demographic and clinical variables of patients with obesity, overweight and normal weight were compared with the F test for continuous variables and the Chi-squared test for categorical variables. Continuous variables are presented as the mean and standard deviation, and categorical variables are presented as frequencies. A binary logistic regression was used to examine which factors were strongly associated with obesity and overweight (with normal weight as the reference group). All *p*-values were 2-tailed, and *p* < 0.05 was the threshold for statistical significance. All statistical analyses were performed with SPSS 21.0.

## Results

### Demographic and clinical characteristics of patients with BD

There were 10,046 inpatients in the Affiliated Brain Hospital of Guangzhou Medical University in 2019, among which 1,426 were BD patients aged ≥18 years. After excluding 257 (18.0%) patients without a recorded BMI, a total of 1169 patients (630 men and 539 women) were included in this study. There were no significant difference in age and gender between patients with BMI and patients without BMI.

The demographic and clinical characteristics of patients with obesity, patients who were overweight and patients with normal weight were compared and are presented in Table 1. The average age of all the included patients was 35.2 ± 13.8 years, ranging from 18 to 79 years. The average disease duration was 9.1 ± 9.3 years, and the average length of hospital stay was 37.5 ± 42.3 days. Among all of the patients, 70 (6.0%) patients had diabetes, and 81 (6.9%) patients had hypertension. Significant differences between the groups were found in the following variables: duration of BD, uric acid, ALT, triglyceride, TC, HDL cholesterol and LDL cholesterol (*p* < 0.05). There were significant differences in the prevalence of sex, diabetes and hypertension among the three groups (*p* < 0.05). Patients with obesity had much higher levels of all metabolic parameters than patients with overweight and normal weight, except for HDL (*p* < 0.05).

**Table 1:** Demographic and clinical characteristics of BD patients with obesity, patients with overweight and patients with normal weight.

	**Obesity**	**Overweight**	**Normal weight**	***X*^2^/F**	***P***
	**(*n*, %)**	**(*n*, %)**	**(*n*, %)**		
Cases	246 (21.0)	377 (32.2)	546 (46.7)	-	-
**Gender**					
Male	140 (22.2)	254 (40.3)	236 (37.5)	53.489	0.001
Female	106 (19.7)	123 (22.8)	310 (57.5)		
Diabetes	25 (10.2)	20 (5.3)	25 (4.6)	9.854	0.007
Hypertension	29 (11.8)	25 (6.6)	27 (4.9)	12.393	0.002
Hyperlipidemia	85 (35.0)	70 (18.8)	61 (11.4%)	61.268	0.001
Age (years)	36.8 ± 12.9	35.4 ± 13.4	34.5 ± 14.5	2.319	0.099
The length of hospital stay (days)	42.7 ± 45.9	36.2 ± 43.7	36.2 ± 39.6	2.301	0.101
Duration (years)	11.9 ± 9.6	9.1 ± 9.3	7.9 ± 9.0	16.455	0.000
Uric acid (μmol/L) (*n =* 1,166)	448.9 ± 136.0	413.9 ± 110.4	361.3 ± 102.8	57.292	0.001
ALT (U/L) (*n =* 1,168)	31.4 ± 25.1	23.4 ± 18.1	20.5 ± 24.0	19.760	0.001
Total cholesterol (mmol/L) (*n =* 1,152)	4.8 ± 1.1	4.6 ± 1.0	4.3 ± 0.8	20.064	0.001
Triglyceride (mmol/L) (*n =* 1,152)	1.6 ± 0.8	1.3 ± 0.7	1.0 ± 0.6	51.237	0.001
HDL cholesterol (mmol/L) (*n =* 1,152)	1.2 ± 0.2	1.2 ± 0.3	1.3 ± 0.3	22.756	0.001
LDL cholesterol (mmol/L) (*n =* 1,152)	2.8 ± 0.9	2.6 ± 0.8	2.3 ± 0.6	37.872	0.001

### The prevalence of obesity and overweight

The overall prevalence of obesity and overweight in patients with BD was 21.0% (246/1169) and 32.2% (377/1169), respectively. A total of 22.2% (140/630) of men and 19.7% (106/539) of women were obese; 40.3% (254/630) of men and 22.8% (123/539) of women were overweight, and this difference was significant (*X*^2^ = 53.489, *p* = 0.001).

### Factors associated with obesity and overweight in patients with BD

As shown in Table 2, after adjusting for relevant variables, the results of a stepwise forward binary logistic regression showed that the duration of BD, uric acid, ALT, triglyceride and LDL cholesterol were significantly associated with obesity (*p* < 0.05).

**Table 2:** Associated factors for obesity in patients with BD.

	***P***	**OR**	**95% CI**
Age	0.053	0.982	0.965–1.000
Duration of BD	<0.001	1.058	1.033–1.084
Male	0.317	0.820	0.555–1.210
The length of hospital stay	0.195	1.003	0.999–1.007
Diabetes	0.867	0.937	0.438–2.002
Hypertension	0.192	0.604	0.283–1.288
Uric acid	<0.001	1.004	1.003–1.006
ALT	0.010	1.010	1.002–1.017
Total cholesterol	0.413	0.784	0.437–1.405
Triglyceride	<0.001	1.745	1.289–2.363
HDL cholesterol	0.189	0.553	0.229–1.337
LDL cholesterol	0.023	2.180	1.114–4.268

### Factors associated with overweight in patients with BD

As shown in Table 3, a stepwise forward binary logistic regression suggested that male sex and uric acid were significantly associated with overweight (*p* < 0.05).

**Table 3:** Associated factors for overweight in patients with BD.

	***P***	**OR**	**95% CI**
Age	0.675	0.997	0.984–1.011
Duration of BD	0.112	1.015	0.997–1.034
Male	<0.001	2.060	1.509–2.812
The length of hospital stay	0.704	0.999	0.996–1.003
Diabetes	0.881	1.056	0.518–2.153
Hypertension	0.578	0.826	0.420–1.622
Uric acid	<0.001	1.003	1.001–1.004
ALT	0.637	0.998	0.992–1.005
Total cholesterol	0.533	1.200	0.676–2.131
Triglyceride	0.197	1.194	0.912–1.563
HDL cholesterol	0.251	0.628	0.284–1.389
LDL cholesterol	0.692	1.144	0.589–2.222

## Discussion

Obesity is a major risk factor for non-communicable diseases in the general population and among people with mental disorders. To the best of our knowledge, this is the first and largest sample-based study investigating the prevalence and associated factors of obesity and overweight in patients with BD in China. We found that the prevalence rates of obesity and overweight were 21.0% and 32.2% in 1,169 in patients with BD, respectively. Given the global trend of increased obesity in the general population and the high prevalence of obesity in people with mental disorders, it is not surprising that obesity and overweight were prevalent in our study. The prevalence of obesity was 31.9% in 2007 and 37.2% in 2017 among two large-sample nationally representative surveys in the Chinese general population, which was much higher than our findings (22). However, the total prevalence of overweight and obesity was 52.2% in 2007 and 58.0% in 2017 in that study, which is similar to the findings (53.2%) in our study. The mean age in that study was 44.8 years in 2007 and 43.8 years in 2017, both much higher than our study (35.2 years). Age is an independent risk factor for obesity (22). Another study in China with 441 thousand adults reported that people aged 45–54 years had the highest frequency of overweight and obesity (23). A meta-analysis with 101 studies including 698,905 participants identified that the prevalence of obesity increased with age, and people aged > 40 years had the highest percentage of obesity and overweight in Middle East countries (24). The lower mean age in our study may account for our lower prevalence of obesity. We found that overweight and obesity were prevalent, which is consistent with existing studies of people with BD (15). However, our 21.0% prevalence rate of obesity is lower than the 29.0% reported in a recent meta-analysis (15), similar to a previous study in China (20). That study with a smaller sample noted that the prevalence of obesity and overweight were 17.74 and 47.58% in 124 Chinese stable BD patients, respectively (20). The discrepancy between our study and other studies in the prevalence of overweight and obesity among BD patients may be due to differences in race, region, quality of health care, lifestyles, dietary habits, genetic factors, criteria for obesity, stage of BD, and psychotropic use.

We found that the duration of BD was significantly associated with obesity, which is consistent with the findings of a previous study among patients with BD at the initiation of the acute phase treatment (25). Some studies also found that the duration of BD was associated with a higher BMI (26), increased medical burden (27), worsening of both the clinical profile and brain structural alterations (28), metabolic syndrome (29), and diabetes (30). Recently, a positive correlation between the duration of BD and the number of mood episodes with both hypertension and the 10-year cardiovascular risk score has been found in patients with BD type I (31). A longer duration of BD may be linked with mood instability, chronicity, unhealthy lifestyles, and more complex psychopharmacological treatments involving antipsychotics, which may lead to insulin resistance, metabolic syndrome, and autonomic nervous system dysfunction, increasing the risk of developing obesity (31).

Conflicting results regarding gender differences in the prevalence of overweight and obesity have been reported in the general population and people with BD (15, 32). Between 1975 and 2014, the global age-standardized prevalence of obesity more than tripled in men (from 3.2 to 10.8%) and doubled in women (from 6.4% to 14.9%), and the global prevalence of morbid obesity and severe obesity was much higher in women than in men (32). However, according to a systematic analysis for the global burden of disease study in 2013, gender differences in the levels and trends of overweight and obesity were much smaller in both developed and developing countries (33). In addition, a meta-analysis showed that there were no sex differences in the prevalence of obesity in BD (15). Two studies suggested that male BD patients had a higher prevalence of obesity and overweight than female BD patients (25, 34), and we found that male BD patients had a higher prevalence of obesity and overweight and that male sex was significantly associated with overweight in our study. Interestingly, a study in the Chinese general population also found that men had higher rates of obesity and overweight than women in 2007 and 2017 (22). We speculate that different behavioral styles (excessive eating, drinking and smoking) may account for the higher prevalence of obesity and overweight in men with BD.

Uric acid is the end product of the purinergic system, and hyperuricemia has been recognized as a potentially treatable risk factor for cardiometabolic diseases; it could predict the development of hypertension, metabolic syndrome, type 2 diabetes, coronary artery disease, left ventricular hypertrophy, atrial fibrillation, myocardial infarction, stroke, heart failure and chronic kidney disease in the general population (35). In addition, hyperuricemia can accelerate hepatic and peripheral lipogenesis to cause obesity (36). People with BD have a high prevalence of hyperuricemia, with a figure ranging between 27.7 and 31.91% (37, 38). Increasing evidence from genetic and clinical studies suggests that purinergic system dysfunction may play a role in the pathophysiology and therapeutics of BD (39), and increased uric acid levels are correlated with impulsivity, excitatory behavior, irritability, a hot temperament and severe manic symptoms in patients with BD. Moreover, hyperuricemia was associated with metabolic parameters in people with BD (37, 40). For example, a study in Italy reported that metabolic syndrome, abdominal circumference and triglyceride levels had a significant effect on uric acid in patients with BD (40). Hyperuricemia has also been found to be associated with metabolic syndrome and a larger waist circumference in Chinese patients with BD (37). A significant relationship between uric acid and a higher prevalence of obesity and overweight was found in the present study, which is consistent with previous studies (36). Our findings suggest that more studies investigating the potential association between uric acid and obesity should be conducted among patients with BD.

ALT is the liver enzyme most strongly correlated with liver fat accumulation and has been found to be closely related to obesity and metabolic syndrome (41). For example, a large sample-based study with 3,843 pediatric and adolescent subjects suggested that each unit increment in ALT elevated the odds of being metabolically unhealthy obese by 2% compared with metabolically healthy non-obese individuals (42). In a study with 5,411 adolescents aged 12–19 in the US, ALT levels were significantly correlated with BMI Z score and metabolic syndrome Z score (*p* < 0.0001) (43). A longitudinal study also indicated that weight gain was significantly associated with an increased risk of elevated ALT levels (44). Elevated ALT was also associated with natural death in BD (45). In line with these studies, we found that ALT was significantly associated with obesity in patients with BD.

We found that triglycerides and LDL cholesterol were associated with increased obesity in BD, consistent with previous studies showing an association between obesity and blood lipid-related parameters in the general population (46). Triglycerides were much higher in drug-naïve BD patients and BD patients taking medications than in healthy controls, and triglycerides were found to be associated with cognitive dysfunction and worse cognitive flexibility in BD (47). Triglycerides were the most accurate factor to identify individuals with greater cognitive impairment from among patients with severe mental disorders (48). In the general population, triglycerides are an independent risk factor for cardiovascular disease (49), and could serve as an independent marker for an increased risk of cardiovascular diseases in patients with type 2 diabetes mellitus (50). Furthermore, triglycerides are dose-dependently associated with increased risks of cardiovascular diseases and all-cause mortality in the general population (51). Notably, elevated triglycerides and other metabolic risk factors are highly prevalent yet under-treated in patients with BD (52).

There were several limitations in our study. First, a major limitation is its retrospective and naturalistic design, which cannot prove a direct causal association between any variables and obesity in patients with BD. Second, we only included inpatients with BD from a psychiatric hospital, and outpatients and patients in general hospitals and communities were not included. This population could not represent all patients with BD in China. Third, data on other important associated factors of obesity and overweight were not available, such as smoking, a family history of obesity, the type and duration of psychotropic medicine, physical activity, genetic factors and dietary habits. Not including these factors could limit our study, and these associated factors should be considered in future studies. Fourth, healthy controls matched for age and sex were not included in this study, so we could not compare obesity and overweight in patients with BD and the general population. Fifth, ~20% of BD patients with missing BMI were excluded, which might cause potential selection bias. On the other hand, its large sample size is a strength of our study.

## Conclusion

In summary, this is the first study to investigate the prevalence and associated factors of obesity and overweight in a relatively large sample of adult patients with BD in China. The present study provided detailed features of obesity and overweight in Chinese patients with BD. In this retrospective and cross-sectional study, obesity and overweight were prevalent among inpatients with BD. In a binary logistic regression analysis, duration of BD and the levels of uric acid, ALT, triglycerides, and LDL cholesterol were identified as predictors for the occurrence of obesity, whereas male sex and uric acid level were associated with a higher frequency of overweight. The results of the present study indicate a need to implement early screening, prevention and interventions for obesity and overweight in patients with BD in China.

## Data availability statement

The original contributions presented in the study are included in the article/supplementary material, further inquiries can be directed to the corresponding authors.

## Ethics statement

The studies involving human participants were reviewed and approved by Ethics Committee of the Affiliated Brain Hospital of Guangzhou Medical University. Written informed consent for participation was not required for this study in accordance with the national legislation and the institutional requirements.

## Author contributions

WY, HW, RL, HL, ZS, SS, and YZ designed the study and wrote the protocol. WY, HW, RL, HL, and ZS were collected data. WY and HW wrote the first draft of the manuscript. All authors contributed to the article and approved the submitted version.

## Conflict of interest

The authors declare that the research was conducted in the absence of any commercial or financial relationships that could be construed as a potential conflict of interest.

## Publisher's note

All claims expressed in this article are solely those of the authors and do not necessarily represent those of their affiliated organizations, or those of the publisher, the editors and the reviewers. Any product that may be evaluated in this article, or claim that may be made by its manufacturer, is not guaranteed or endorsed by the publisher.
